# WNT5A signaling impairs breast cancer cell migration and invasion via mechanisms independent of the epithelial-mesenchymal transition

**DOI:** 10.1186/s13046-016-0421-0

**Published:** 2016-09-13

**Authors:** Chandra Prakash Prasad, Shivendra Kumar Chaurasiya, William Guilmain, Tommy Andersson

**Affiliations:** 1 Cell and Experimental Pathology, Department of Translational Medicine, Lund University, Clinical Research Centre, Skåne University Hospital, SE-20502 Malmö, Sweden; 2 Present Address: Department of Applied Microbiology, School of Biological Sciences, Dr HS Gour Central University, Sagar, Madhya Pradesh India

**Keywords:** WNT5A, Human mammary epithelial cells, Breast cancer, EMT, ERK1/2, CD44, AKT, Cell migration and invasion

## Abstract

**Background:**

WNT5A (-/-) mammary tissue has been shown to exhibit increased ductal elongation, suggesting elevated mammary cell migration. Increased epithelial cell migration/invasion has often but not always been linked to the epithelial-mesenchymal transition (EMT). In the current study, we investigated the loss of WNT5A in HB2 human mammary epithelial cells and hypothesized that this loss increased their invasion via the EMT. Based on these results, we postulated that suppression of breast cancer cell migration and invasion by WNT5A is due to EMT reversal.

**Methods:**

WNT5A was transiently knocked down using specific siRNAs, whereas WNT5A signaling was induced in MDA-MB468 and MDA-MB231 breast cancer cells by stably transfecting cells with WNT5A or treating them with recombinant WNT5A (rWNT5A). Changes in EMT markers, CD44, pAKT and AKT expression were assessed using Western blotting and immunofluorescence. The physiological relevance of altered WNT5A signaling was assessed using migration and invasion assays.

**Results:**

WNT5A knockdown in HB2 mammary epithelial cells resulted in EMT-like changes and increased invasiveness, and these changes were partially reversed by the addition of rWNT5A. These data suggest that WNT5A might inhibit breast cancer cell migration and invasion by a similar EMT reversal. Contrary to our expectations, we did not observe any changes in the EMT status of breast cancer cells, either after treatment with rWNT5A or stable transfection with a WNT5A plasmid, despite the parallel WNT5A-induced inhibition of migration and invasion. Instead, we found that WNT5A signaling impaired CD44 expression and its downstream signaling via AKT. Moreover, knocking down CD44 in breast cancer cells using siRNA impaired cell migration and invasion.

**Conclusions:**

WNT5A bi-directionally regulates EMT in mammary epithelial cells, thereby affecting their migration and invasion. However, the ability of WNT5A to inhibit breast cancer cell migration and invasion is an EMT-independent mechanism that, at least in part, can be explained by decreased CD44 expression.

**Electronic supplementary material:**

The online version of this article (doi:10.1186/s13046-016-0421-0) contains supplementary material, which is available to authorized users.

## Background

The Wnt family of proteins includes secreted lipid-modified glycoproteins that regulate diverse cellular processes, such as proliferation, differentiation, apoptosis and migration [1, 2]. To date, 19 members of the Wnt gene family have been identified and placed into three different classes based on their transforming potential [3]. WNT5A belongs to the non-transforming class and elicits non-canonical (β-catenin-independent) signaling upon binding to different receptors or co-receptor complexes (Frizzled, RYK, ROR1/2, LRP5/6 and CD146) [4, 5].

WNT5A signaling has been shown to be essential for normal development [6]. In the context of mammary gland development, WNT5A signaling plays an important role in ductal branching, and its loss can induce increased ductal extensions and larger terminal buds [7]. These findings suggest that WNT5A functions to limit mammary epithelial cell migration during development.

The loss of WNT5A signaling plays an important role in breast cancer progression, thereby making WNT5A a tumor suppressor. Moreover, the loss of WNT5A protein in breast cancer patients has been associated with poor prognosis, early recurrence and reduced overall survival [8–11]. Using immunohistochemical (IHC) staining, Borcherding et al. reported strong WNT5A expression in normal mammary ducts and acini, but reduced WNT5A expression was observed in 75 % of triple negative breast cancers (TNBC), an aggressive breast cancer subtype [12]. Accordingly, a recent study by Zhong et al. demonstrated a loss of WNT5A in 60 % of TNBC patients, and this loss positively correlated with lymph node metastasis and poor recurrence-free survival (RFS) [13]. Additionally, we have also demonstrated that WNT5A expression can be used as a potential prognostic biomarker in pre-menopausal patients with ER+ breast tumors [14]. Other studies of breast cancer cells involving the treatment of cells with either recombinant WNT5A (rWNT5A) or the stable transfection of WNT5A protein demonstrated that breast cancer cell adhesion increased, whereas their migration decreased [15–17].

A key biological event responsible for tumor cell invasion and metastasis is the epithelial-mesenchymal transition (EMT). Cancer cells undergoing EMT show a loss of cell-cell contact and polarity and acquire migratory and invasive properties [18, 19]. Recent studies have highlighted the importance of WNT5A in the regulation of the EMT. In colon cancer, where WNT5A acts as a tumor suppressor, WNT5A restoration has been shown to inhibit EMT by antagonizing the Wnt/β-catenin signaling pathway [20]. In a similar study, Cai et al. demonstrated that microRNA-374a directly suppresses multiple negative regulators of Wnt/β-catenin signaling, including WNT5A, and this suppression induces EMT in breast cancer [21]. These studies also suggest that canonical Wnt/β-catenin signaling plays a parallel role as a potential regulator of EMT in these cancers. In contrast, WNT5A has been shown to positively drive EMT-like changes in gastric cancer, pancreatic cancer, ovarian cancer and malignant melanomas, where WNT5A acts as a tumor promoter [22–26].

The aim of the present study was to clarify whether the loss of WNT5A expression can induce an EMT phenotype to consequently increase the invasiveness of breast epithelial cells. Moreover, this study aimed to determine whether inhibition of such a mechanism could explain our previous observation that WNT5A can suppress the invasion of breast cancer cells. In the present study, we employed HB2 human mammary epithelial cells (which endogenously express WNT5A) as a model system to elucidate the early events associated with the loss of WNT5A protein and EMT changes. However, we also examined whether WNT5A inhibits breast cancer cell migration/invasion via a reversal of EMT in breast cancer cells.

## Methods

### Cell lines

The human mammary epithelial cell line HB2, which is a subclone of the MTSV1-7 cell line, was kindly provided by the laboratory of Dr. J. Taylor Papadimitriou (ICRF, UK). HB2 cells are a suitable model to study mammary cell responses because they endogenously express WNT5A protein (mimicking the clinical situation; normal mammary epithelial cells and acini endogenously express WNT5A protein). Thus, the modulation of WNT5A expression can be directly investigated via phenotypic changes and changes in the expression of biomarkers/molecules of interest in these cells. The human mammary carcinoma cell lines MDA-MB468 (Lot No. 60880170) and MDA-MB231 (Lot No. 61093072) were procured directly from the American Type Culture Collection (ATCC). All cell lines were grown in DMEM (Hyclone, UT, USA) supplemented with 10 % FBS, 5 U/ml penicillin, 0.5 U/ml streptomycin, and 2 mM glutamine. The growth media for HB2 cells was also supplemented with 10 μg/ml bovine insulin and 5 μg/ml hydrocortisone. The cell lines were frequently screened for mycoplasma contamination using an EZ-PCR kit (HaEmek, Israel).

### siRNA transfections

Sequence-specific siRNAs were used to transiently knock down WNT5A, E-cadherin and CD44. We purchased Silencer® select pre-designed siRNAs for WNT5A [ID: s14871 (siRNA 1) and ID: s14872 (siRNA 2)], E-cadherin (ID: 146381) and negative control #1 siRNA from Applied Biosystems (CA, USA). SMARTpool CD44 siRNA (h) (Cat. no. sc-29342) was procured from Santa Cruz Biotechnology, Inc. (TX, USA). Briefly, cells were transiently transfected with a transfection complex consisting of 50 or 100 nM siRNA (final concentration) and Lipofectamine 2000 transfection reagent (Invitrogen, CA, USA) suspended in serum-free DMEM. After 6 h, the transfection complex was removed, and the cells were replenished with fresh DMEM containing 10 % FBS. The cells were then allowed to grow for the next 48 h prior to being used for Western blotting, immunofluorescence staining, or an invasion assay.

### Western blotting

Untreated cells or cells treated with siRNAs, rWNT5A, or U0126 were washed with ice-cold PBS and lysed in ice-cold phosphorylation lysis buffer (PLB). The estimation of protein concentration, SDS-PAGE and visualization procedures were performed as described in Prasad et al. [16]. The following primary antibodies were used: anti-WNT5A from R & D Systems (MN, USA); anti-E-cadherin, anti-pAKT (ser473), anti-total AKT, anti-vimentin, anti-N-cadherin, anti-Slug and anti-pERK1/2 from Cell Signaling Technology (MA, USA); anti-α-tubulin antibodies from Santa Cruz Biotechnology, Inc. (TX, USA); and anti-CD44 (156-3C11) antibody from Thermo Scientific (CA, USA). The secondary antibodies used in this study were goat anti-mouse, goat anti-rabbit and rabbit anti-goat, which were procured from Dako (Glostrup, Denmark).

### Phase-contrast and confocal microscopy

For phase-contrast microscopy, HB2 cells were grown in 6-well culture plates or on glass coverslips and treated with either WNT5A siRNA or E-cadherin siRNA. For the negative control, cells were treated with scrambled siRNA. After 48 h, the HB2 cells were photographed (using a Nikon model TMS-F microscope) and analyzed with NIS-Elements software (Tokyo, Japan).

For confocal microscopy, the cells were grown on 13-mm glass coverslips for 24 h at 37 °C in a humidified incubator under 5 % CO_2_. For the rWNT5A rescue experiments, cells were transfected with WNT5A siRNA transfection for 48 h and subsequently treated with rWNT5A (0.4 μg/ml) for another 24 h. For CD44 immunostaining of breast cancer cells stably expressing WNT5A protein (MDA-MB468-5A and MDA-MB231-5A), the cells were allowed to grow on 13-mm glass coverslips for 48 h at 37 °C in a humidified incubator under 5 % CO_2_ in reduced serum conditions. After the treatments, the cells were washed with PBS, fixed with 4 % paraformaldehyde for 20 min and permeabilized using 0.1 % Triton X-100 for 3 min (all steps were performed at RT). Subsequently, the cells were washed with PBS and incubated with the appropriate primary antibody (i.e., E-cadherin, vimentin or CD44) in a humidified chamber at 4 °C. After an overnight incubation, the cells were washed and incubated with the appropriate secondary antibody (either goat anti-mouse or goat anti-rabbit Alexa 488) (Molecular Probes™, Oregon, USA) for 45 min at room temperature. After extensive washing, the cells were counterstained with DAPI (300 nM in PBS) for 2 min. Next, the coverslips were mounted on glass slides with Dako fluorescent mounting medium and viewed with a Carl Zeiss LSM 700 (Oberkochen, Germany) confocal microscope. Immunofluorescence staining was semi-quantitatively assessed using ImageJ software (NIH, MD, USA). Briefly, the images were first processed using the process tool to sharpen the images, and plot profiles were generated utilizing a line cross-sectioning approach for each cell. The areas inside the peaks depicting membranous or cytoplasmic intensities were evaluated using the freehand selection and measurement tools. The resultant graphs reveal the mean fluorescence (a.u.) corresponding to the membranous or cytoplasmic staining of the protein in each cell.

### Transwell invasion and migration assays

WNT5A, E-cadherin or CD44 siRNA-transfected cells were washed and detached with Versene (Gibco®, NY, USA) and subsequently re-suspended as single-cell suspensions in low-serum DMEM (supplemented with 1 % FBS). The cell invasion assays were carried out in BD Matrigel™ invasion chambers (MA, USA), and the cells were counted using an automated cell counter (Countess™, Invitrogen, Korea). A total of 50,000 cells in low-serum DMEM were added to the upper chamber [for rWNT5A treatment, rWNT5A (0.4 μg/ml) was freshly added to the medium], and the lower chamber was filled with 0.7 ml of DMEM supplemented with 10 % FBS. The cells were allowed to invade for 24 h at 37 °C in a humidified incubator containing 5 % CO_2_. After incubation, the cells in the insert were fixed with 4 % paraformaldehyde for 10 min at RT. The non-invading cells on the upper side of the insert were removed with a cotton-tipped applicator and further processed as described by Linnskog et al. [27]. Transwell migration assays for CD44 siRNA-treated breast cancer cells were performed in a similar manner using a cell culture insert containing a PET membrane with an 8.0 μm pore size (Ref# 353097; Corning Incorporated, NY, USA).

### Wound healing assay

The migration of MDA-MB468 and MDA-MB231 cells was analyzed using a wound-healing assay. A total of 2 × 10^5^ cells/well were seeded in 12-well tissue culture plates and grown either in the presence of rWNT5A (0.4 μg/ml) or the vehicle control suspended in low-serum DMEM (supplemented with 1 % FBS) for 48 h. The rWNT5A was replenished every 24 h. Wounds were then created by scratching the monolayer, and the cells were subsequently allowed to migrate for 24 h under the same media conditions. The wounds were photographed at 10X magnification with a Nikon microscope (Model: TMS-F) immediately after wounding and 72 h after wounding. The images were compared and analyzed with ImageJ software (NIH, MD, USA).

### BrdU incorporation assay

The proliferation of MDA-MB468 and MDA-MB231 cells was examined for 72 h using an ELISA BrdU kit from Roche Diagnostics GmbH (Mannheim, Germany). Briefly, 1 × 10^4^ cells/well were seeded in a 96-well ELISA plate and treated for 72 h with either rWNT5A (0.4 μg/ml) or the vehicle control suspended in low serum DMEM (supplemented with 1 % FBS). The rWNT5A was replenished every 24 h. During the final 24 h, 10 μM BrdU was added to the cells. After the termination of the experiment, the cells were fixed with FixDenat for 30 min and incubated with anti-BrdU-POD for 90 min. Antibody binding was detected via the addition of a specific substrate, and the resultant absorbance was recorded according to the manufacturer’s instructions.

### Statistical analysis

All the data presented herein are expressed as the mean ± standard error of the mean. Each experiment was repeated at least three times unless otherwise stated. A statistical analysis was performed using Student’s t-test, and *p* values <0.05 were considered significant. All statistical tests and graphs were generated using GraphPad Prism 5.0 software (CA, USA).

## Results

### siRNA-mediated knockdown of WNT5A induces “EMT-like” changes in human mammary epithelial HB2 cells

The experiments in this study were conducted because the levels of WNT5A protein were previously shown to be higher in the pre-neoplastic mammary gland and early tumors than in late-stage tumors [12]. Thus, we hypothesized that the loss of WNT5A in non-cancerous breast cells is associated with changes in the EMT status of cells. To investigate this hypothesis, we used human mammary epithelial HB2 cells in this study [28] because they are non-cancerous and endogenously express WNT5A protein. Recently, Nash et al. advocated the use of luminal HB2 over basal MCF-10A cells for a 3D multi-cellular in vitro model of normal human breast tissue because the morphology attained by HB2 cells in tri-culture was similar to that of normal human breast acini [29]. In addition, two breast cancer cell lines, MDA-MB468 and MDA-MB231 cells, were examined in this study.

**Fig. 1:**
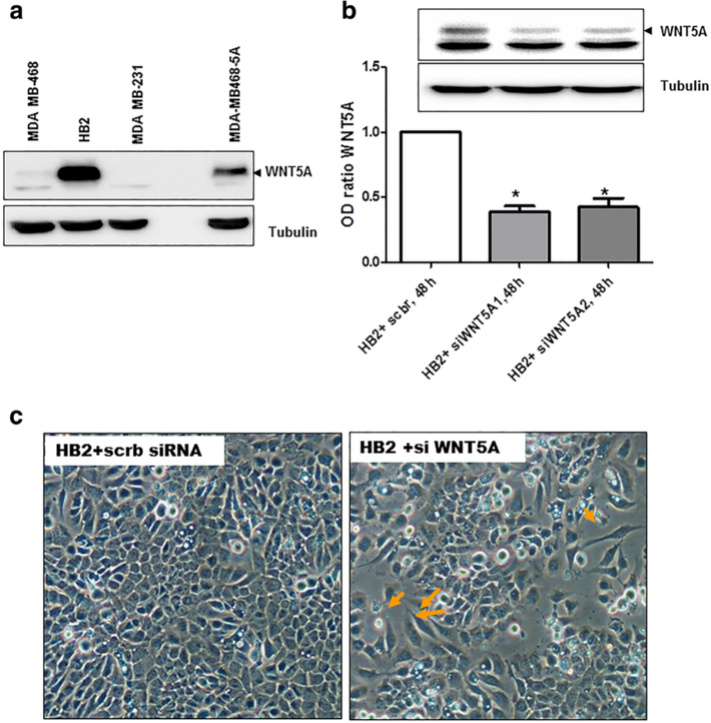
The loss of WNT5A induces “EMT-like” changes in human mammary epithelial HB2 cells. **a** Representative Western blot showing the presence of WNT5A protein in whole-cell lysates from MDA-MB468, HB2 and MDA-MB231 cells (*n* = 3). MDA-MB468 cells that were stably transfected with the WNT5A plasmid (MDA-MB468-5A) were used as a positive control for the experiment. Tubulin was used as a loading control. **b** Two different anti-WNT5A siRNA oligonucleotides were tested on HB2 cells. Total cell lysates were prepared 48 h after transfection, and Western blotting for the WNT5A protein was performed (as described in the Methods section). Error bars represent the standard error of the mean (*n* = 4). *p < 0.05. **c** Phase-contrast microscopy showing morphological changes in HB2 cells grown on glass coverslips and transfected with WNT5A siRNA for 48 h. The magnification used was 20X

**Fig. 2:**
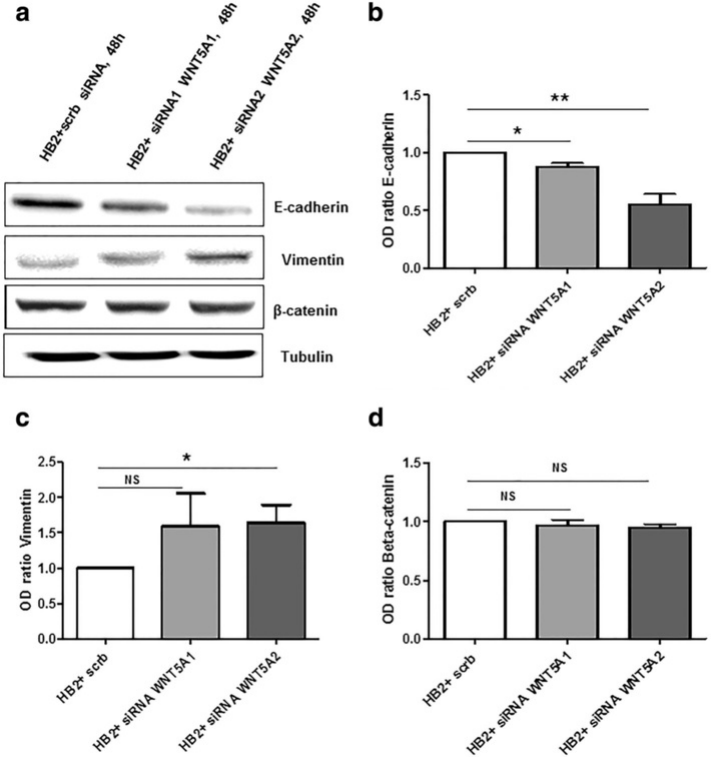
The loss of WNT5A in HB2 cells induces changes in EMT markers. **a** Representative Western blots showing changes in EMT markers upon the treatment of HB2 cells with WNT5A siRNAs. Two sequence-specific siRNAs targeted against WNT5A were used in the present experiment (as described in the Methods section). The EMT markers **b** E-cadherin, **c** vimentin and **d** β-catenin were quantified by calculating the integrated densitometric values and normalizing them to the tubulin levels. Statistical comparisons were made with Student’s t-test (**b**, **c** and **d**). All error bars represent the standard error of the mean (*n* = 5). *p < 0.05, **p = 0.01

In initial experiments, endogenous WNT5A expression was evaluated in all three breast cell lines via a Western blot analysis (Fig. 1a). WNT5A protein expression was not detectable in either breast cancer cell line (MDA-MB468 and MDA-MB231) compared to HB2 cells, which endogenously express WNT5A protein (Fig. 1a). Next, HB2 cells were transiently transfected with two different sequence-specific siRNAs targeting WNT5A (as described in the Methods section) for 48 h, and Western blotting was performed using whole cell lysates to analyze the changes in WNT5A protein expression. The Western blot data demonstrated that transfection with siRNAs targeting WNT5A mRNA significantly decreased the levels of WNT5A protein (Fig. 1b). Moreover, a morphological evaluation of WNT5A siRNA-treated HB2 cells revealed distinct phenotypic changes, such as the loss of cell-cell adhesion, fibroblast-like morphology and cellular scattering (Fig. 1c). These results further prompted us to investigate the changes in EMT markers in WNT5A siRNA-treated HB2 cells. Specifically, transfection with two different sequence-specific WNT5A siRNAs resulted in the deregulation of various EMT markers in HB2 cells (Fig. 2a). Integrated densitometric values (IDVs) revealed a significant decrease in the expression of the epithelial marker E-cadherin (Fig. 2b) and an increase in the expression of the mesenchymal marker vimentin (Fig. 2c) in WNT5A siRNA-treated HB2 cells compared with controls. However, the levels of β-catenin did not change (Fig. 2d). Overall, our results clearly demonstrate that WNT5A is integral to the maintenance of epithelial architecture in mammary epithelial HB2 cells. Subsequent experiments employed only WNT5A siRNA 2 because the results from the knockdown showed that this siRNA produces more consistent and reproducible results than WNT5A siRNA 1.

### Addition of rWNT5A rescues the “EMT-like” changes induced by WNT5A knockdown in HB2 cells

**Fig. 3:**
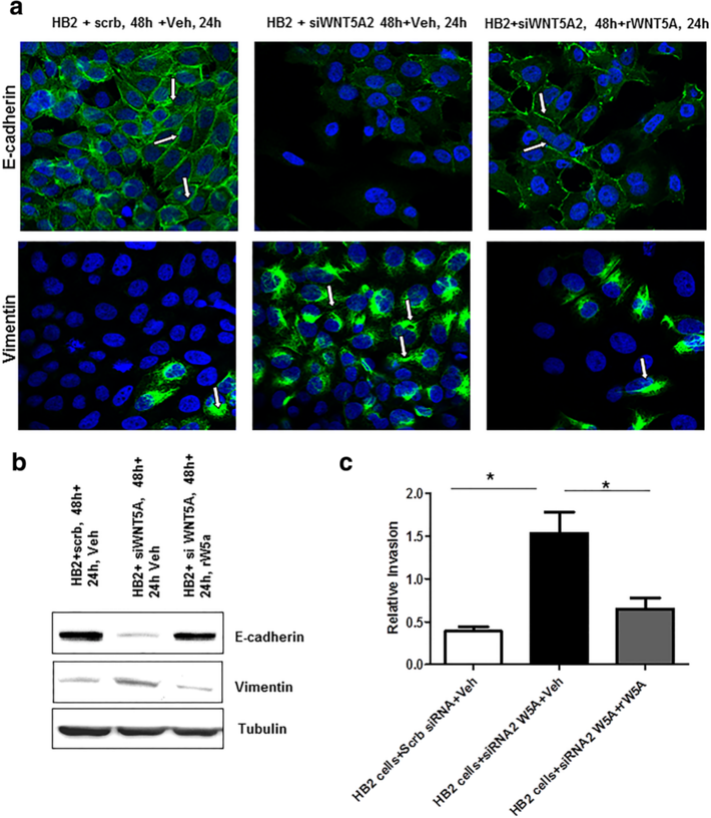
rWNT5A partially rescues the EMT changes induced in HB2 cells. **a** Immunofluorescence staining for E-cadherin and vimentin was performed for the WNT5A rescue experiments. In these experiments, HB2 cells that were transiently transfected with WNT5A siRNA were treated with rWNT5A (0.4 μg/ml). For visualization, a secondary goat anti-mouse/-rabbit Alexa-488-labeled antibody was used together with DAPI counterstaining. The magnification used was 60X (*n* = 3). **b** Representative Western blot demonstrating a reversal in the expression of the epithelial markers E-cadherin and vimentin in rWNT5A-treated cells compared with cells treated with WNT5A siRNA alone. **c** An invasion analysis was performed using a Transwell assay, in which WNT5A siRNA-treated cells were plated on Transwell invasion inserts in either the presence or absence of rWNT5A. Vehicle-exposed cells were used as negative controls. Statistical comparisons were made with Student’s t-test. All error bars represent the standard error of the mean (*n* = 4). *p < 0.05

In the next set of experiments, we investigated the ability of ectopic WNT5A to reverse the “EMT-like” characteristics of HB2 cells transfected with WNT5A siRNA. For these experiments, we used recombinant WNT5A (rWNT5A) (R&D Systems, MN, USA). HB2 cells that had been transiently transfected with WNT5A siRNA 2 for 48 h were treated with rWNT5A (0.4 μg/ml dose) for 24 h. In the control experiments, vehicle (0.1 mM EDTA, 0.5 % CHAPS, and 0.5 % BSA dissolved in PBS) was used instead of rWNT5A. An immunofluorescence analysis revealed that membranous E-cadherin expression was drastically decreased in HB2 cells treated with WNT5A siRNA and partially restored after rWNT5A addition (Fig. 3a*panel: E-cadherin*). In agreement with this observation, we also observed an increase in the mesenchymal protein vimentin in WNT5A siRNA-treated HB2 cells; this effect was subsequently reduced by rWNT5A (Fig. 3a*panel: vimentin*). The immunofluorescence data were further validated by semi-quantitatively estimating the fluorescence intensities of membranous E-cadherin and vimentin in rWNT5A-treated and untreated HB2 cells (Additional file 1a and b). Similar to the immunofluorescence data, Western blotting of the whole cell lysate also revealed an increase in E-cadherin expression and a decrease in vimentin expression after the rWNT5A treatment of siRNA-transfected HB2 cells (Fig. 3b). The treatment of WNT5A siRNA HB2 cells with rWNT5A also significantly decreased the expression of other mesenchymal markers (i.e., N-cadherin and Slug) compared to their increased expression in WNT5A siRNA-treated HB2 cells (Additional file 1c, d and e). As previously observed, we did not find any changes in the β-catenin expression profile in rWNT5A-treated and untreated WNT5A siRNA-transfected HB2 cells (*data not shown*).

To assess the effect the above-described changes in EMT markers on HB2 cell physiology, we conducted invasion assays (as described in the Methods section). We observed that WNT5A-depleted HB2 cells (transfected with WNT5A siRNA) were more invasive (*p* = 0.011) than scrambled control-treated cells, suggesting that the EMT changes associated with a loss of WNT5A in HB2 cells were coupled with increased invasion (Fig. 3c). Furthermore, we also observed that the exogenous supplementation of rWNT5A significantly inhibited this increased invasion (*p* = 0.035) (Fig. 3c). These data corroborate the changes in EMT marker profiles and suggest that the loss of WNT5A triggers mammary cells to acquire a more invasive and mesenchymal phenotype.

As a proof-of-concept, we specifically knocked down the expression of E-cadherin in HB2 cells to demonstrate that a decrease in this epithelial marker can result in the increased invasion of HB2 cells (Additional file 2a) and that this decrease is an important event in WNT5A siRNA-treated HB2 cells. Our results also showed that the effect of treating HB2 cells with E-cadherin siRNA on morphology is similar to that of WNT5A siRNA treatment (Additional file 2b). Moreover, further analysis revealed that the loss of E-cadherin alone cannot regulate the expression of the vimentin and WNT5A proteins in HB2 cells (Additional file 2c).

Overall, these data suggest that the loss of WNT5A stimulates mammary epithelial HB2 cells towards EMT and increased invasion; however, the subsequent restoration of WNT5A resulted in EMT reversal and impaired invasion.

### WNT5A-treated breast cancer cells exhibit no change in EMT markers

**Fig. 4:**
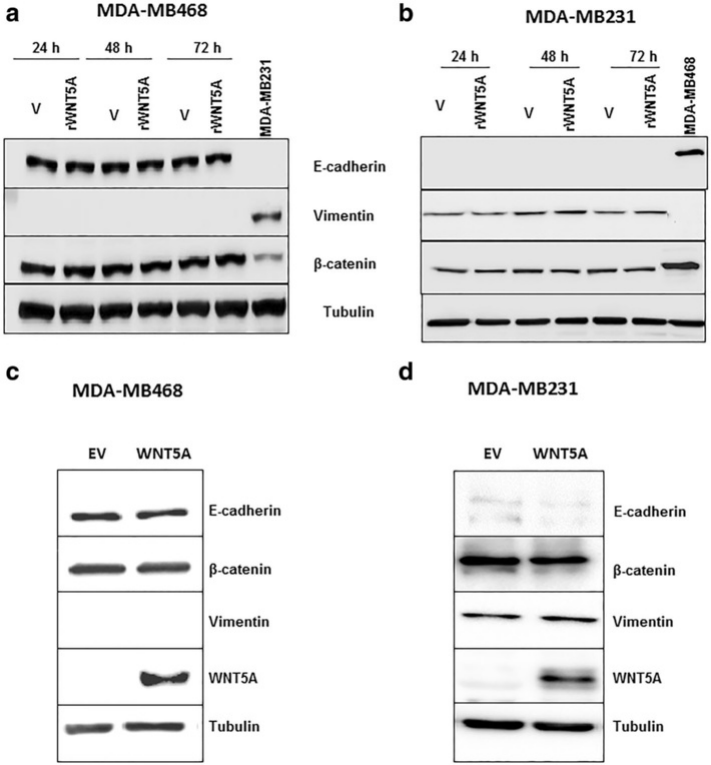
The WNT5A-mediated effects on breast cancer cells were independent of EMT reversal. **a** Representative Western blot showing changes in EMT markers (viz. E-cadherin, vimentin and β-catenin) in MDA-MB468 cells after exposure to rWNT5A for 24, 48 and 72 h. **b** Representative Western blots showing changes in EMT markers (viz. E-cadherin, vimentin and β-catenin) in MDA-MB231 cells after exposure to rWNT5A for 24, 48 and 72 h. **c** Representative Western blot showing changes in EMT markers in WNT5A-expressing MDA-MB468 cells compared with empty vector (EV)-transfected MDA-MB468 cells. **d** Representative Western blot showing changes in EMT markers in WNT5A-expressing MDA-MB231 cells compared with empty vector (EV)-transfected MDA-MB231 cells

In the current study, we found that WNT5A loss in human mammary epithelial HB2 cells induced EMT and that ectopic WNT5A treatment can reverse the EMT process. These results prompted us to speculate that a similar EMT reversal mediates the ability of WNT5A signaling to impair breast cancer cell migration and invasion. To investigate this possibility, we used the MDA-MB468 and MDA-MB231 metastatic breast cancer cell lines. Because both cell lines lack endogenous WNT5A expression, the cells were stimulated with rWNT5A (0.4 μg/ml) for 24, 48 and 72 h, and total protein was then extracted from the cells for Western blotting (as described in the Methods section). In MDA-MB468 cells, the Western blot analysis revealed no changes in the protein expression of any EMT marker (E-cadherin, vimentin or β-catenin) relative to the vehicle-treated control when the cells were treated with rWNT5A for 72 h (Fig. 4a). MDA-MB468 cells did not express vimentin protein. We further validated these results with an RT-qPCR analysis for these EMT markers at 24, 48 and 72 h and did not find changes in any analyzed transcript (*data not shown*). Similar results were obtained in MDA-MB231 cells treated with rWNT5A for 72 h: treatment with rWNT5A did not cause changes in the protein levels (Fig. 4b) or the mRNA levels (*data not shown*) of EMT markers.

Consequently, we hypothesized that long-term exposure to WNT5A is required to induce EMT reversal in breast cancer cells. To investigate this possibility, we analyzed the changes in EMT marker status in MDA-MB468 and MDA MB-231 cells stably transfected with a WNT5A plasmid (MDA-MB468-5A and MDA-MB231-5A cells) relative to MDA-MB468 and MDA-MB231 cells stably transfected with an empty vector (MDA-MB468-EV; MDA-MB231-EV). We did not observe changes in any EMT marker analyzed at the protein level (Fig. 4c and d). Notably, we have previously demonstrated that breast cancer cells transfected with the WNT5A plasmid exhibit low migration and invasion compared to control MDA-MB-468-EV cells [16].

### WNT5A inhibits the migration and invasion of breast cancer cells via an EMT-independent mechanism(s)

**Fig. 5:**
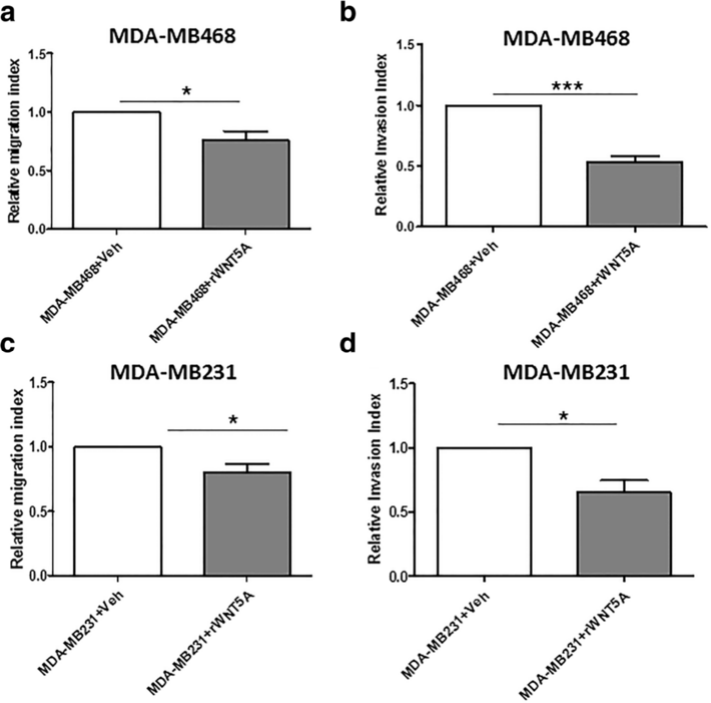
WNT5A significantly decreases the migration and invasion of breast cancer cells. **a** A wound-healing migration assay and **b** a Transwell invasion assay were performed with the breast cancer cell line MDA-MB468 in either the presence or absence of rWNT5A (0.4 μg/ml) for 72 h. Vehicle-treated MDA-MB468 cells were used as controls. Similarly, the relative migration and invasion (**c** and **d**) of the breast cancer cell line MDA-MB231 were quantified in the presence or absence of rWNT5A (0.4 μg/ml) for 72 h. Vehicle-treated MDA-MB231 cells were used as controls. Statistical comparisons were made with Student’s t-test (**a**, **b**, **c**, and **d**). All error bars represent the standard error of the mean (*n* = 4). *p < 0.05, ***p = 0.001

WNT5A-treated breast cancer cells did not exhibit changes EMT markers, which led us to hypothesize that WNT5A suppresses the migration and invasion of breast cancer cells via an EMT-independent mechanism(s). To investigate this possibility, we performed a 72 h wound healing migration assay using MDA-MB468 and MDA-MB231 cells in the presence of rWNT5A, as described in the Methods section. This experiment was vital because in our previous studies, we only investigated the effect of rWNT5A on cancer cell migration/invasion for 24–48 h. In agreement with our earlier studies, we found that the migration of both breast cancer cell lines was significantly inhibited compared with the controls when treated with rWNT5A (0.4 μg/ml) for 72 h (Fig. 5a and c). We also performed a parallel BrdU proliferation assay at the same time points to eliminate the possibility that the changes in migration were due to an indirect effect on proliferation (Additional file 3). In the next step, the invasion potential of each breast cancer cell line (MDA-MB468 and MDA-MB231) was analyzed. We found a significant reduction in the invasion of both breast cancer cell lines after treatment with rWNT5A for 72 h (Fig. 5b and d). These results further confirmed that WNT5A inhibits the migration and invasion of breast cancer cells without reversing the EMT process.

### WNT5A inhibits CD44-AKT signaling in breast cancer cells

**Fig. 6:**
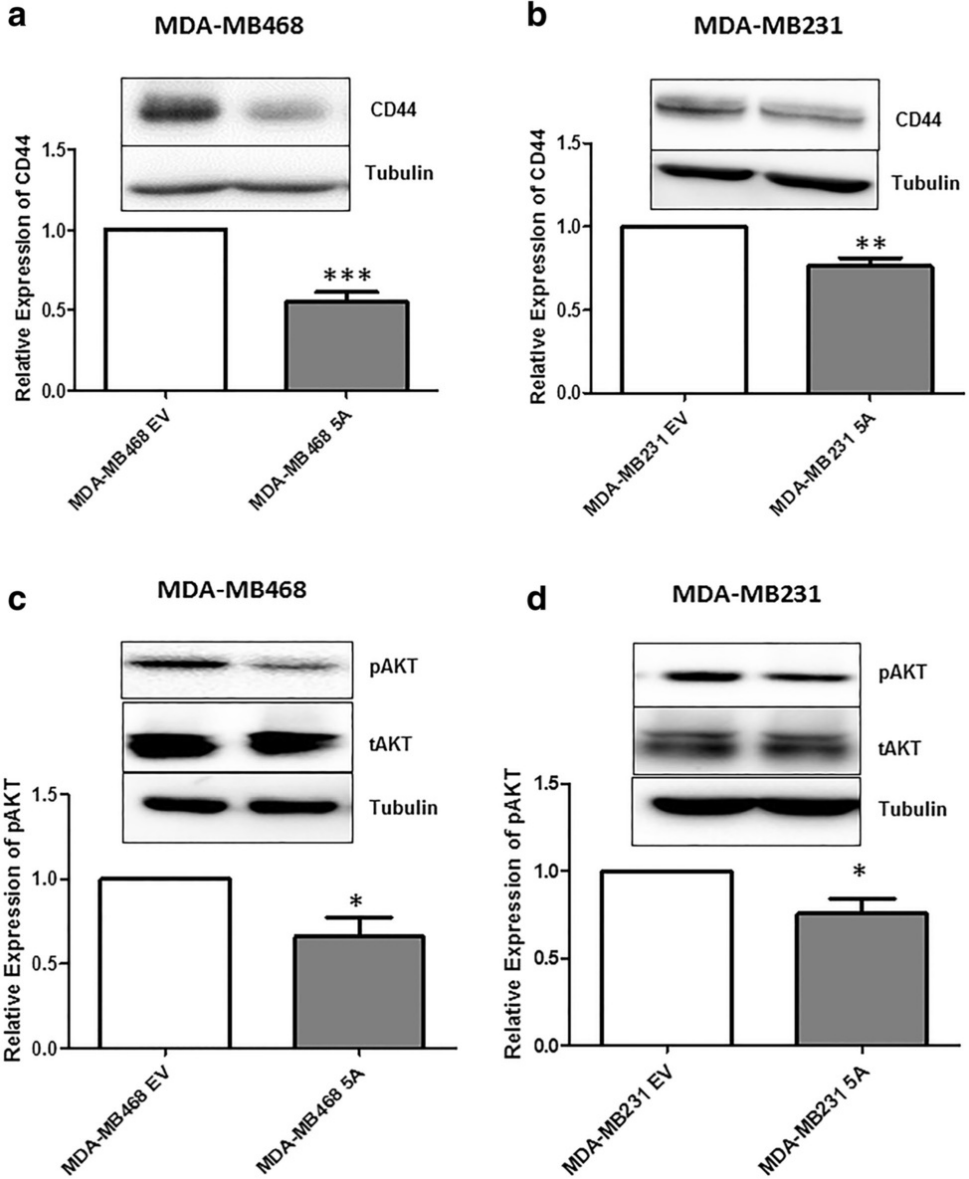
WNT5A significantly inhibits CD44 and pAKT expression in breast cancer cells. **a** CD44 expression was analyzed in MDA-MB468-EV cells and MDA-MB468-5A cells by Western blotting. The CD44 levels in MDA-MB468-EV and MDA-MB468-5A cells were quantified by calculating integrated density values and normalizing them to the tubulin levels. **b** CD44 expression was analyzed in MDA-MB231-EV cells and MDA-MB231-5A cells by Western blotting. CD44 levels in MDA-MB231-EV and MDA-MB231-5A cells were quantified by calculating integrated density values and normalizing them to tubulin levels. pAKT activation was analyzed in MDA-MB468-EV cells and MDA-MB468-5A cells as in panel **c** and in MDA-MB231-EV cells and MDA-MB231-5A cells as in panel **d**. pAKT levels were quantified by calculating integrated density values and normalizing them to total AKT levels. Statistical comparisons were made with Student’s t-test (**a**, **b**, **c** and **d**). All error bars represent the standard error of the mean (*n* = 5). *p < 0.05, **p = 0.01, ***p = 0.001

Because the WNT5A-mediated inhibition of breast cancer cell migration and invasion was independent of EMT reversal, we explored the involvement of alternative pathways that might be triggered by WNT5A to curb the spread of breast cancer cells. To this end, we investigated the effect of WNT5A on CD44, a transmembrane glycoprotein, for several reasons. First, increased CD44 expression has been correlated with breast cancer cell metastasis and a poor prognosis [30, 31]. Second, Jiang et al. combined microarray and RNA-Seq approaches to demonstrate that WNT5A alters the splicing of CD44 and that the levels of specific variant isoforms of CD44 (e.g., CD44v4 and CD44v6) were reduced in a WNT5A-expressing cell line [17]. In our previous studies, we also demonstrated that the role of WNT5A in increased adhesion is mediated by its ability to phosphorylate DDR1, resulting in the reduced migration of breast cancer cells [32, 33]. Because CD44 overexpression alone is sufficient to induce breast cancer cell migration and invasion [34], we investigated the ability of WNT5A signaling to directly regulate CD44 expression in breast cancer cells. Therefore, we next analyzed the expression of CD44 protein in breast cancer cells stably expressing WNT5A protein. CD44 immunofluorescence staining revealed that both breast cancer cell lines (MDA-MB468-5A and MDA-MB231-5A) expressed reduced levels CD44 membrane protein compared to their counterpart controls, which were transfected with empty vector (Additional file 4). Subsequently, we validated our findings by Western blotting, demonstrating that WNT5A expression does indeed inhibit the expression of CD44 protein in WNT5A-expressing breast cancer cell lines (Fig. 6a and b). CD44 is a receptor that can stimulate tumorigenic signals via a variety of downstream pathways and effectors, such as PI3K/AKT signaling and MMP-9. Therefore, we next investigated whether the WNT5A-mediated reduction in CD44 decreased PI3K/AKT signaling in breast cancer cells. We found a significant reduction in the expression of pAKT in both WNT5A-transfected breast cancer cell lines (MDA-MB468-5A and MDA-MB231-5A) compared to control cells (Fig. 6c and d). The data were further validated in parental MDA-MB231 cells treated with rWNT5A (0.2 and 0.4 μg/ml) for 24 h (Additional file 5a). We observed a dose-dependent decrease in the expression of pAKT, thereby validating our finding that WNT5A indeed inhibits the CD44-AKT signaling pathway in breast cancer cells.

Furthermore, to assess the effect of WNT5A on CD44 protein expression in HB2 mammary epithelial cells, we knocked down the expression of WNT5A these cells using siRNA (Additional file 5b). This knockdown did not affect the level of CD44 protein, suggesting that WNT5A does not modulate CD44 expression in HB2 mammary epithelial cells.

### CD44 knockdown reduces the level of pAKT, migration and invasion of breast cancer cell

**Fig. 7:**
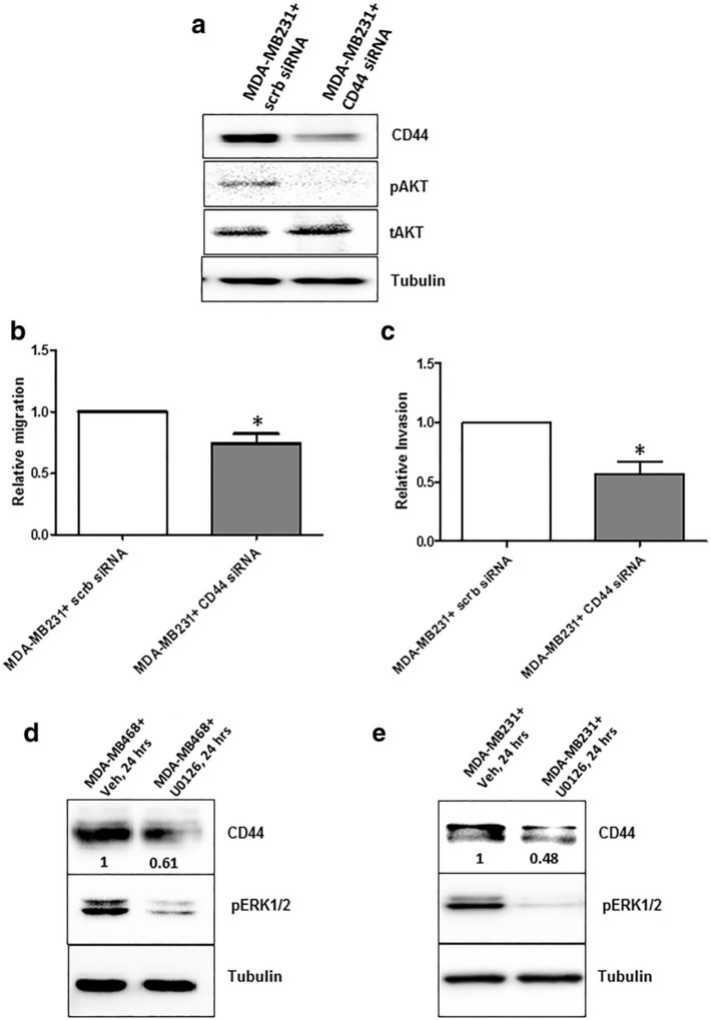
CD44 and ERK1/2 inhibition and the migration and invasion of MDA-MB231 breast cancer cells. MDA-MB231 cells were transiently transfected with CD44 siRNA (as described in the Methods section), and changes in the cellular characteristics were investigated. **a** Representative Western blot demonstrating that the silencing of CD44 expression induces changes in pAKT levels. After CD44 knockdown, MDA-MB231 cells were subjected to **b** Transwell migration and **c** Transwell invasion assays. Statistical comparisons were made with Student’s t-test (**b** and **c**). All error bars represent the standard error of the mean (*n* = 4). *p < 0.05. **d** MDA-MB468 and **e** MDA-MB231 breast cancer cells were treated with U0126 (10 μM) or *left* untreated for 24 h, and Western blotting was used to determine CD44 expression. The expression of pERK1/2 was also analyzed as an experimental control. The blots shown are representative of three independent experiments (*n* = 3)

Our experiments showed that WNT5A inhibited CD44 expression in breast cancer cells but not in HB2 cells. Therefore, we hypothesized that reduced levels of CD44 are responsible for the WNT5A-mediated reduction in the migration and invasion of breast cancer cells. To investigate this hypothesis, we used siRNA to knock down CD44 expression in MDA-MB231 cells and evaluated the effect of this knockdown on the pAKT levels, cell migration and invasion. CD44 siRNA-treated cells demonstrated reduced pAKT levels (Fig. 7a) and significant decreases in their migratory and invasive capabilities compared with their respective controls (Fig. 7b and c). In support of these findings, MDA-MB468 cells treated with CD44 siRNA also showed a significant decrease in migration compared to control cells (Additional file 6). Overall, our results demonstrate that the WNT5A-mediated reduction in CD44-AKT signaling plays an important role in inhibiting breast cancer cell migration and invasion.

We previously demonstrated that WNT5A inhibits ERK1/2 signaling in breast cancer cells [16]. In the present study, we showed that WNT5A affects breast cancer cell migration and invasion by inhibiting CD44 expression and downstream AKT signaling. To investigate the role of altered ERK1/2 signaling in the WNT5A-mediated inhibition of CD44, we treated breast cancer cell lines with U0126 (a selective inhibitor of MAP kinase). Treatment with U0126 for 24 h drastically reduced CD44 protein expression in MDA-MB468 (Fig. 7d) and MDA-MB231 (Fig. 7e) cells, suggesting that CD44 is a downstream target of the MAP kinase pathway in these cells. Thus, these results suggest that WNT5A-impaired ERK1/2 signaling, which as mimicked by the ERK1/2 inhibitor U0126 in the present study, reduces CD44 expression in breast cancer cells.

## Discussion

We have previously shown that the loss of WNT5A in HB2 cells leads to cell scattering, reduced cell-collagen interaction and increased motility, and we established the role of WNT5A signaling in regulating DDR1 phosphorylation, increased adhesion and decreased migration [33]. In the present study, knocking down WNT5A in mammary HB2 cells resulted in increased invasion and EMT-like changes. These changes included phenotypic changes, such as cellular scattering, the loss of cell-cell adhesion and a fibroblast-like morphology, as well as significant increases in the expression of established mesenchymal markers. Interestingly, our data revealed that ectopic stimulation with rWNT5A reversed the increased invasion and the EMT-like changes in mammary epithelial HB2 cells.

The above observations corroborate clinical observations made in breast cancer tissues, where a loss of WNT5A expression has been correlated with increased metastasis and poor outcome in breast cancer patients. According to recent findings from Borcherding et al., early breast tumors and pre-neoplastic lesions express WNT5A protein; however, its expression decreases in late-stage tumors and lung metastases [12]. Accordingly, Jönsson et al. have also demonstrated the loss of WNT5A protein in invasive ductal carcinoma patients, which is in contrast to the expression of WNT5A in DCIS (ductal carcinoma in situ) [8]. The current literature on WNT5A expression in breast cancer strongly advocates its role as an oncosuppressor, and the loss of WNT5A expression is one of the causes of increased breast cancer progression [35].

These data suggest that the ability of WNT5A signaling to impair breast cancer cell migration and invasion [16, 17] is due to an EMT reversal similar to that observed in mammary HB2 cells. We previously reported that WNT5A at least in part inhibits the migration and invasion of breast cancer cells by reducing the production and secretion of MMP9 [16]. This finding is relevant to the potential of WNT5A to reverse EMT in breast cancer cells because MMPs have been demonstrated to facilitate EMT in these cells [36]. However, neither the rWNT5A treatment of nor stably transfecting the WNT5A protein into breast cancer cells produced any changes in EMT markers, although WNT5A signaling still inhibited the migration and invasion of both breast cancer cell lines under similar conditions. Thus, WNT5A inhibits the migration and invasion of breast cancer cells via an EMT-independent mechanism. This effect of WNT5A appears to be tumor type specific because a recent study of HCT116 colon cancer cells has suggested that WNT5A can reverse EMT by antagonizing canonical Wnt/β-catenin signaling in these cells [20]. Conversely, we did not find any changes in the β-catenin expression levels in WNT5A siRNA-treated HB2 cells or in breast cancer cell lines exposed to WNT5A treatments. This finding further supports the hypothesis that the function of WNT5A depends on the cancer cell type.

EMT reversal is clearly not responsible for the WNT5A-mediated inhibition of breast cancer cell migration and invasion; therefore, we sought alternative explanations for this phenomenon. Our results demonstrate that WNT5A signaling affects the expression of the transmembrane glycoprotein CD44, and WNT5A is known to alter the splicing of CD44 and its variant isoforms in breast cancer [17]. We clearly showed that WNT5A suppresses CD44, which consequently reduces downstream AKT signaling in breast cancer cells. CD44 is known to regulate PI3K/AKT signaling in breast cancer [37, 38], and this pathway plays an important role in cell survival, invasion and metastasis [39, 40]. Considerable research has indicated that MAPK-ERK signaling regulates CD44 expression in various types of cancer [41–44]. This finding is interesting because we have previously demonstrated that WNT5A inhibits ERK1/2 activity in breast cancer cells [16], which suggests that WNT5A regulates CD44 via ERK1/2. Using an ERK1/2 inhibitor to mimic the inhibition of ERK1/2 activity by WNT5A, we confirmed a link between ERK1/2-MAPK signaling and CD44 expression in breast cancer cells. In accordance with the reported positive association between CD44 and breast cancer metastasis [45–49], the migration and invasion of breast cancer cells significantly impaired in our siRNA-CD44 knockdown experiments.

The experimental data presented herein corroborate the relationship between the expression levels of WNT5A and CD44 in clinical breast cancer samples and their relation to disease-free survival and cumulative survival of patients with breast cancer. Importantly, information on the interaction between WNT5A and CD44 cannot be directly obtained from clinical material, but it can be obtained from experimental data. Therefore, experimental and clinical data are complementary. The data from different research groups have revealed that WNT5A expression in clinical breast cancer samples is marker of good prognosis [8, 12, 14, 35], whereas CD44 expression has been associated with poor prognosis [50]. Similar findings have also been reported in triple-negative and basal breast cancer tissues [13, 51]. Moreover, treatment with either a WNT5A agonist [52] or a monoclonal antibody against CD44 [53, 54] in mouse breast cancer models impaired tumor progression, which suggests a therapeutic possibility. The present study demonstrated that WNT5A signaling can suppress CD44 protein expression and signaling, which supports the use of a WNT5A agonist in the treatment of breast cancer patients.

## Conclusions

Unlike in mammary epithelial cells, the inhibition of migration and invasion by WNT5A signaling is an EMT-independent event in breast cancer cells. This study demonstrated that WNT5A inhibits CD44 expression and its downstream AKT signaling, which further explains how WNT5A signaling impairs breast cancer cell migration and invasion.

## Additional files

Additional file 1: The loss of WNT5A in HB2 cells induces changes in EMT markers and was restored by rWNT5A treatment. Semi-quantification of **a** membranous E-cadherin and **b** Vimentin were performed using ImgaeJ software, as described in the Methods section. For each experiment between 10 and 15 cells were evaluated per slide. All error bars represent the standard error of the mean. **p*<0.05, ***p*=0.01, ****p*=0.001. **c** HB2 cells were transfected with WNT5A siRNA2 for 48 h followed by treatment with rWNT5A (0.4 μg/ml) for 24 h. The cells were lysed, and Western blotting was performed for the N-cadherin and Slug proteins. The quantification of **d** N-cadherin and **e** Slug was performed by calculating the integrated densitometric values and normalizing them to the tubulin levels. Statistical comparisons were made with Student’s t-test (b and c). All error bars represent the standard error of the mean (*n*=4). **p*<0.05. (PDF 405 kb) Additional file 2: The loss of E-cadherin alone can induce ‘EMT-like’ changes. HB2 cells were transiently transfected with E-cadherin siRNA for 48 h, and changes in the cellular characteristics were investigated using **a** a transwell invasion assay, **b** morphological analysis (20X magnification) and **c** Western blotting. HB2 cells treated with scrambled siRNA served as a control. Statistical comparisons were made with Student’s t-test (a). Error bars represent the standard error of the mean (*n*=4). **p*<0.05. (PDF 405 kb) Additional file 3: WNT5A treatment has no effect on breast cancer cell proliferation. Proliferation analysis was carried out using a BrdU incorporation assay (as described in the Methods section). **a** MDA-MB468 and **b** MDA-MB231 cells were treated with rWNT5A for 24, 48 and 72 h. Vehicle-treated cells were used as controls. Statistical comparisons were made with Student’s t-test (a and b). All error bars represent the standard error of the mean (*n*=3). ns=non-significant. (PDF 405 kb) Additional file 4: CD44 immunostaining in WNT5A-expressing breast cancer cell lines. **a** MDA-MB468 and **b** MDA-MB-231 cells transfected with the WNT5A plasmid (MDA-MB468-5A and MDA-MB231-5A) were allowed to grow on 13-mm glass coverslips for 48 h and were then processed for CD44 immunofluorescence staining (as described in the Methods section). The intensity of CD44 expression in WNT5A-expressing cancer cells was compared with empty vector (EV)-transfected control cells (*n*=3). (PDF 405 kb) Additional file 5: WNT5A mediated regulation of CD44 expression in MDA-MB231 and HB2 mammary epithelial cells. **a** Representative western blot showing the expression of CD44, pAKT and total AKT in MDA-MB231 cells treated with rWNT5A (0.2 and 0.4 μg/ml) for 24 h. **b** Human mammary epithelial (HB2) cells were transfected with WNT5A siRNA2 for 48 h, followed by Western blotting for CD44 expression. CD44 was quantified by calculating the integrated densitometric values and normalizing them to the tubulin levels. Statistical comparisons were made with Student’s t-test (b). All error bars represent the standard error of the mean (*n*=3). *ns=non-significant. (PDF 404 kb) Additional file 6: CD44 silencing significantly reduced the migration of MDA-MB468 cells. MDA-MB468 cells were transiently transfected with CD44 siRNA for 48 h (as described in the Methods section) and subjected to transwell migration analysis. Statistical comparisons were made with Student’s t-test. All error bars represent the standard error of the mean (*n*=4). ****p*=0.001. (PDF 405 kb)

## Abbreviations

ECM: Extracellular matrix
EMT: Epithelial mesenchymal transition
MMP: Matrix metalloproteinase
rWNT5A: Recombinant WNT5A
TNBC: Triple negative breast cancer
