# Supplementary figures and images for: WNT5A signaling impairs breast cancer cell migration and invasion via mechanisms independent of the epithelial-mesenchymal transition
Source: J Exp Clin Cancer Res. 2016 Sep 13;35(1):144. doi: 10.1186/s13046-016-0421-0 (PMC5022188; doi:10.1186/s13046-016-0421-0)

Additional File. 1

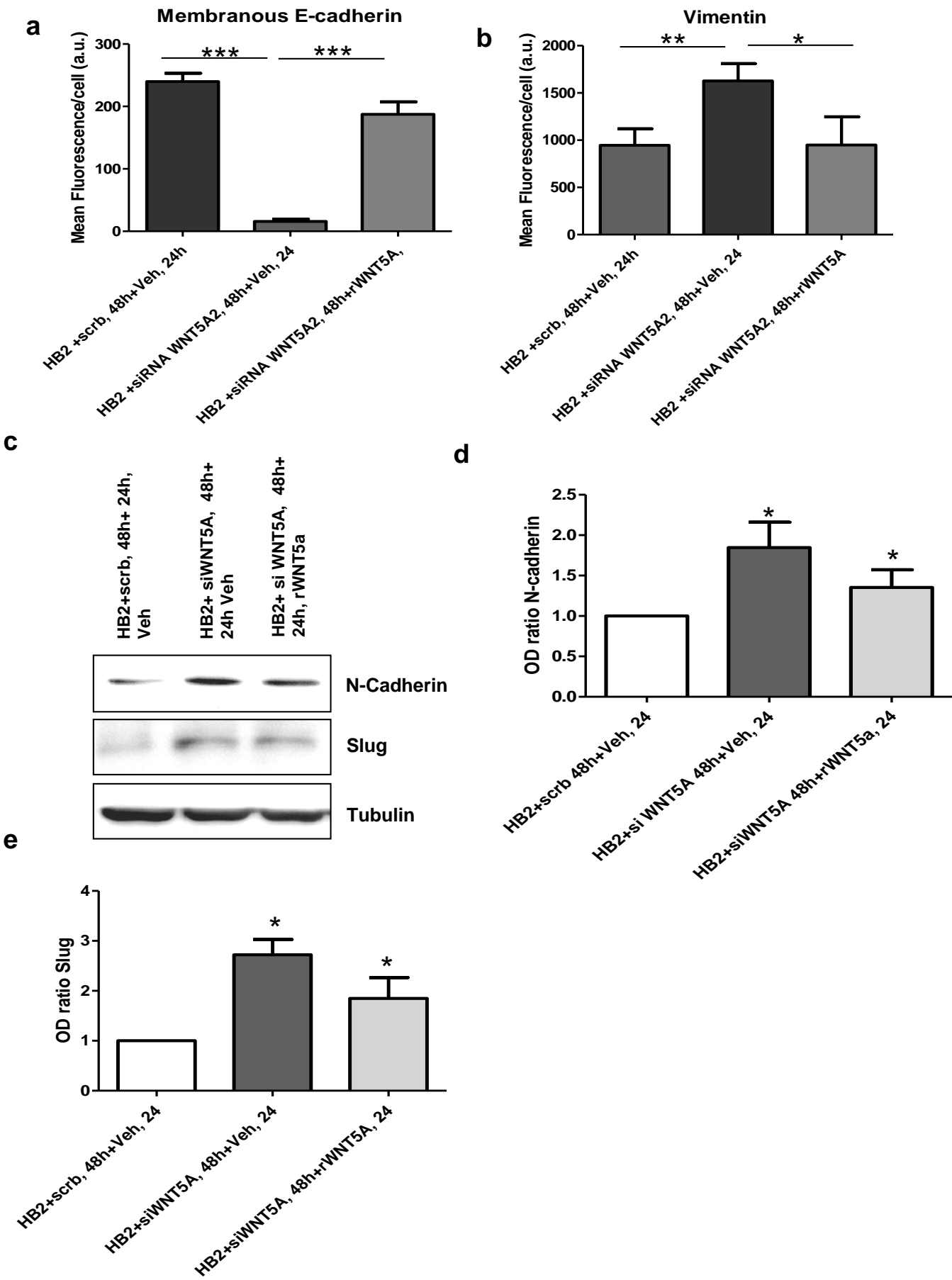

Supplement: Additional file 1: — The loss of WNT5A in HB2 cells induces changes in EMT markers and was restored by rWNT5A treatment. Semi-quantification of a membranous E-cadherin and b Vimentin were performed using ImgaeJ software, as described in the Methods section. For each experiment between 10 and 15 cells were evaluated per slide. All error bars represent the standard error of the mean. *p<0.05, **p=0.01, ***p=0.001. c HB2 cells were transfected with WNT5A siRNA2 for 48 h followed by treatment with rWNT5A (0.4 μg/ml) for 24 h. The cells were lysed, and Western blotting was performed for the N-cadherin and Slug proteins. The quantification of d N-cadherin and e Slug was performed by calculating the integrated densitometric values and normalizing them to the tubulin levels. Statistical comparisons were made with Student’s t-test (b and c). All error bars represent the standard error of the mean (n=4). *p<0.05. (PDF 405 kb) [file 13046_2016_421_MOESM1_ESM.pdf]

a

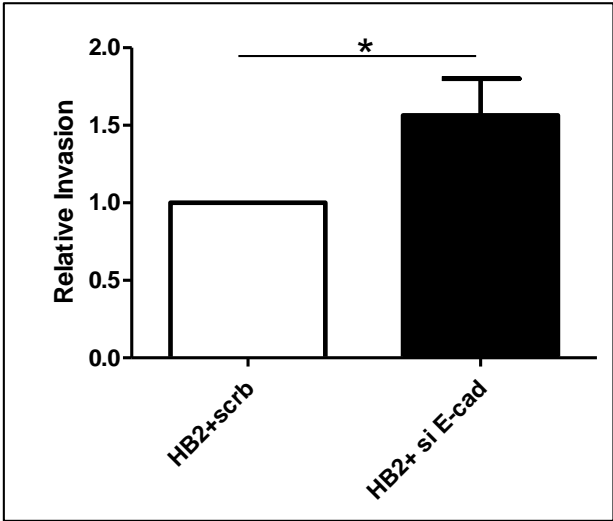

b

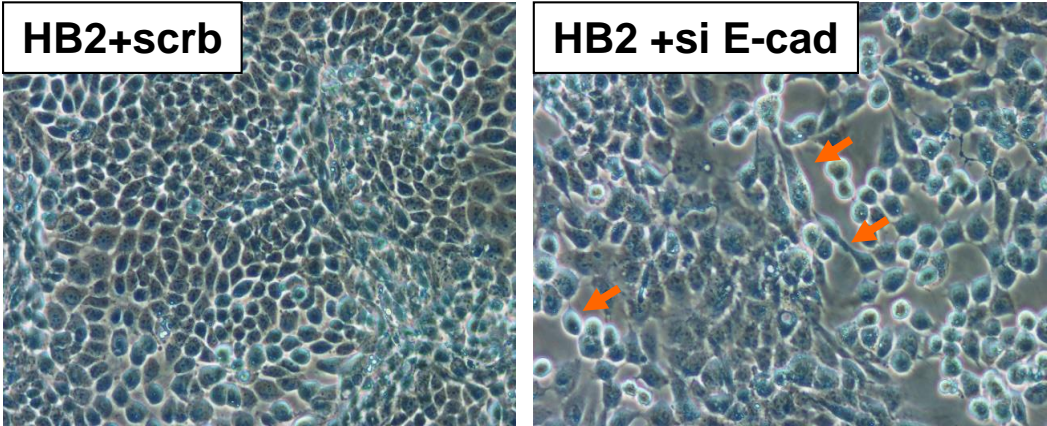

c

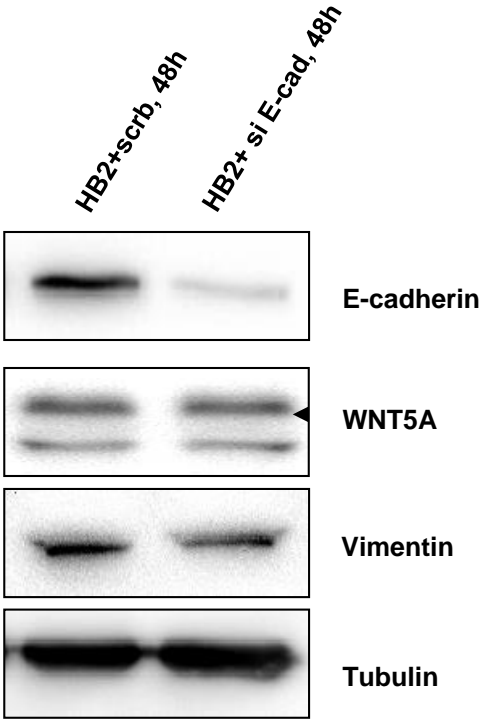

Supplement: Additional file 2: — The loss of E-cadherin alone can induce ‘EMT-like’ changes. HB2 cells were transiently transfected with E-cadherin siRNA for 48 h, and changes in the cellular characteristics were investigated using a a transwell invasion assay, b morphological analysis (20X magnification) and c Western blotting. HB2 cells treated with scrambled siRNA served as a control. Statistical comparisons were made with Student’s t-test (a). Error bars represent the standard error of the mean (n=4). *p<0.05. (PDF 405 kb) [file 13046_2016_421_MOESM2_ESM.pdf]

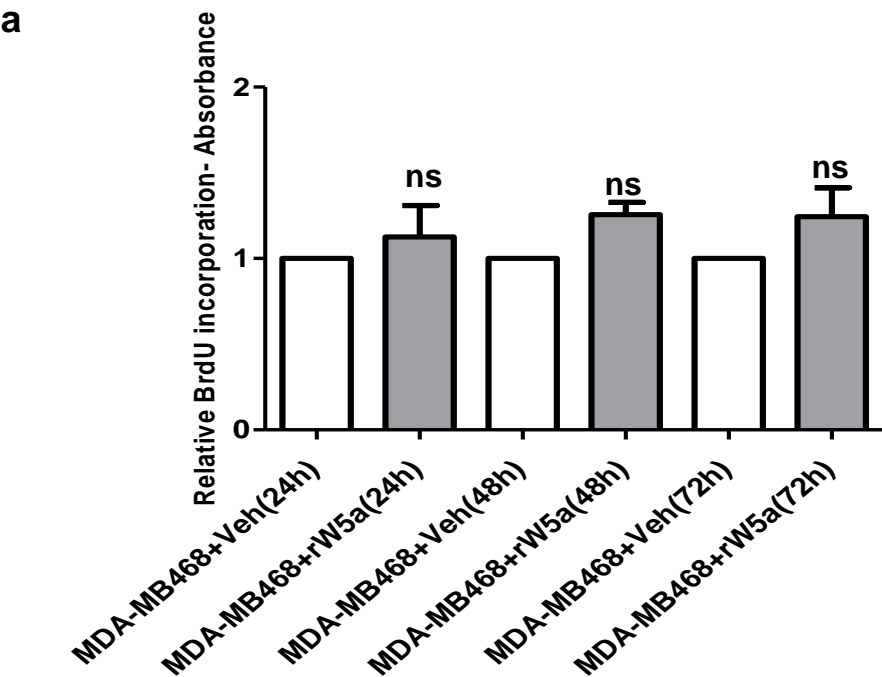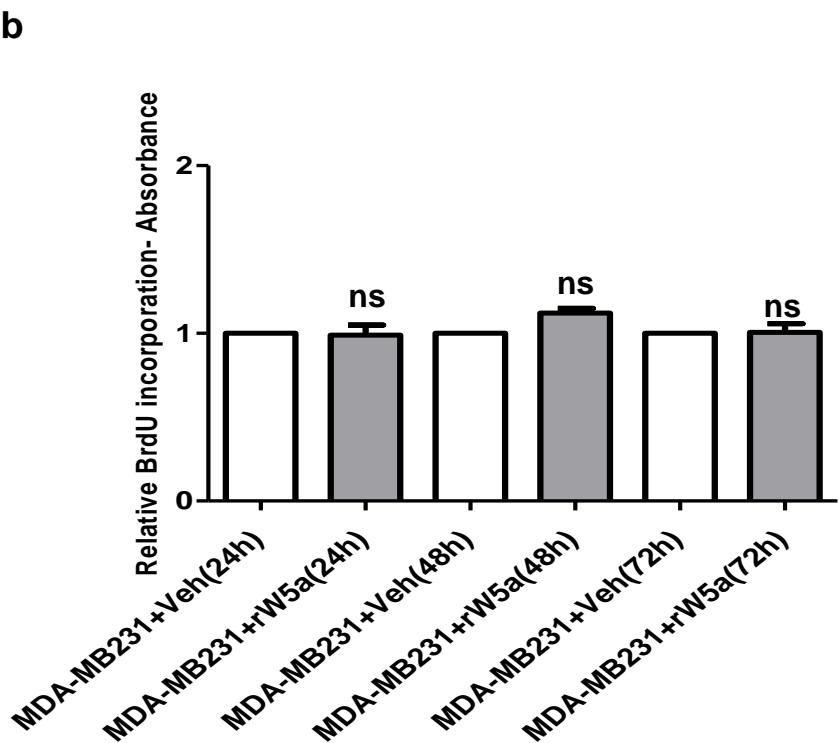

Supplement: Additional file 3: — WNT5A treatment has no effect on breast cancer cell proliferation. Proliferation analysis was carried out using a BrdU incorporation assay (as described in the Methods section). a MDA-MB468 and b MDA-MB231 cells were treated with rWNT5A for 24, 48 and 72 h. Vehicle-treated cells were used as controls. Statistical comparisons were made with Student’s t-test (a and b). All error bars represent the standard error of the mean (n=3). ns=non-significant. (PDF 405 kb) [file 13046_2016_421_MOESM3_ESM.pdf]

a

MDA-MB468-EV

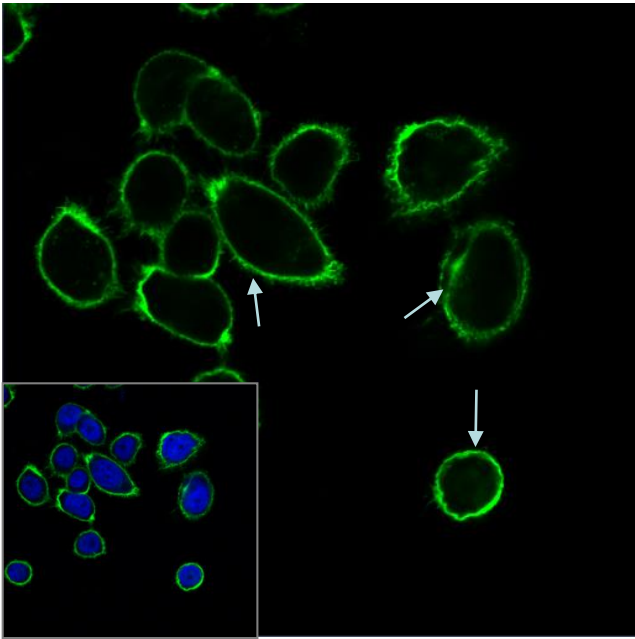

MDA-MB468-5A

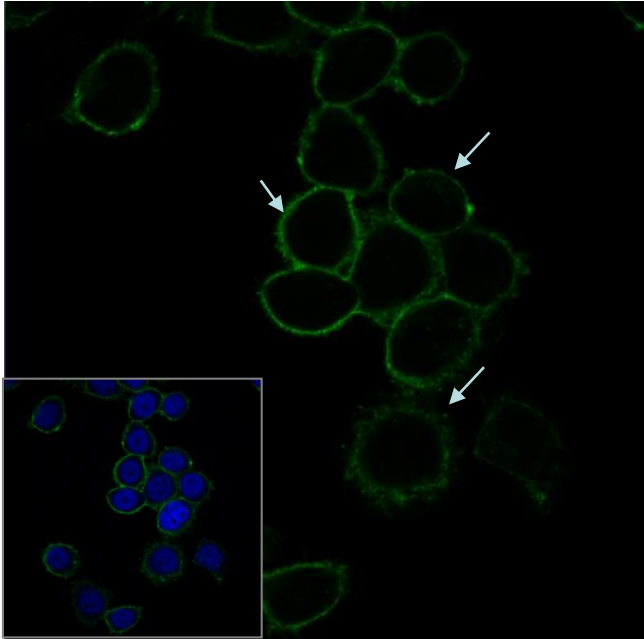

b

MDA-MB231-EV

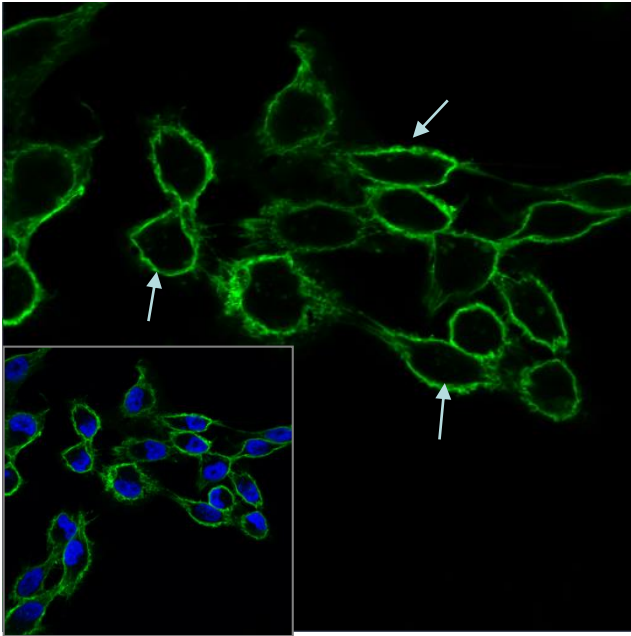

MDA-MB231-5A

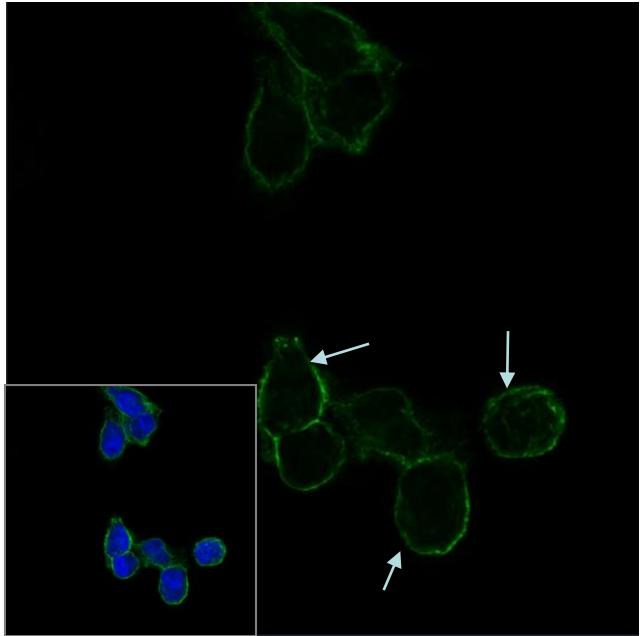

Supplement: Additional file 4: — CD44 immunostaining in WNT5A-expressing breast cancer cell lines. a MDA-MB468 and b MDA-MB-231 cells transfected with the WNT5A plasmid (MDA-MB468-5A and MDA-MB231-5A) were allowed to grow on 13-mm glass coverslips for 48 h and were then processed for CD44 immunofluorescence staining (as described in the Methods section). The intensity of CD44 expression in WNT5A-expressing cancer cells was compared with empty vector (EV)-transfected control cells (n=3). (PDF 405 kb) [file 13046_2016_421_MOESM4_ESM.pdf]

a

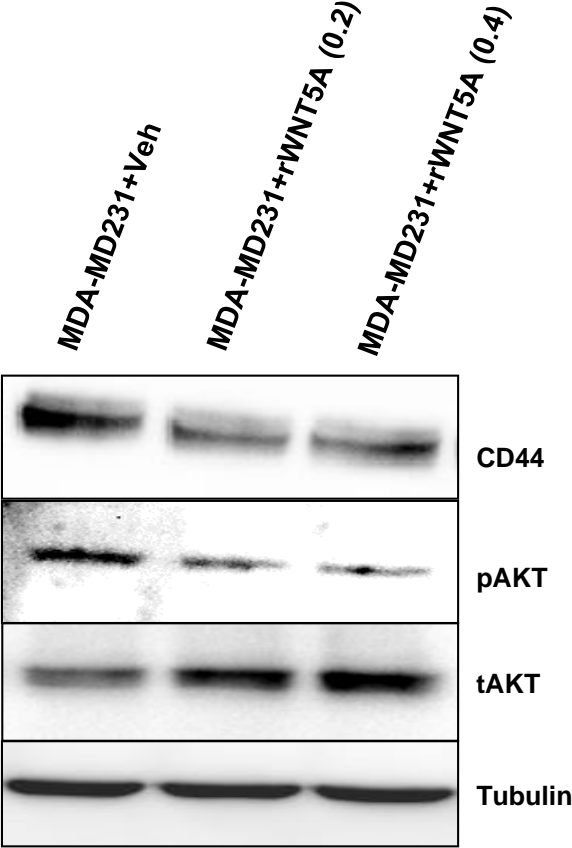

b

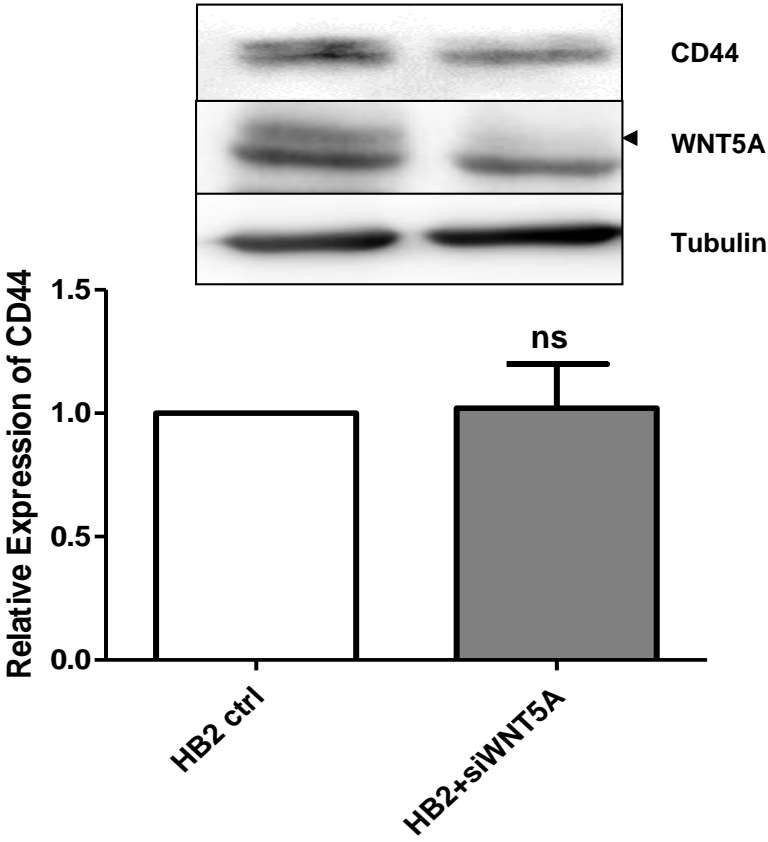

Supplement: Additional file 5: — WNT5A mediated regulation of CD44 expression in MDA-MB231 and HB2 mammary epithelial cells. a Representative western blot showing the expression of CD44, pAKT and total AKT in MDA-MB231 cells treated with rWNT5A (0.2 and 0.4 μg/ml) for 24 h. b Human mammary epithelial (HB2) cells were transfected with WNT5A siRNA2 for 48 h, followed by Western blotting for CD44 expression. CD44 was quantified by calculating the integrated densitometric values and normalizing them to the tubulin levels. Statistical comparisons were made with Student’s t-test (b). All error bars represent the standard error of the mean (n=3). *ns=non-significant. (PDF 404 kb) [file 13046_2016_421_MOESM5_ESM.pdf]

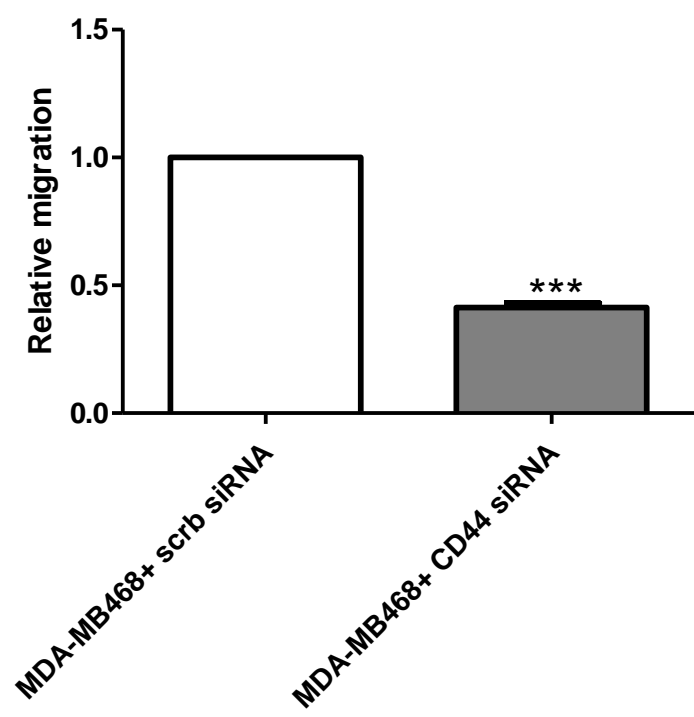

Supplement: Additional file 6: — CD44 silencing significantly reduced the migration of MDA-MB468 cells. MDA-MB468 cells were transiently transfected with CD44 siRNA for 48 h (as described in the Methods section) and subjected to transwell migration analysis. Statistical comparisons were made with Student’s t-test. All error bars represent the standard error of the mean (n=4). ***p=0.001. (PDF 405 kb) [file 13046_2016_421_MOESM6_ESM.pdf]
